# Central Corneal Thickness and Intraocular Pressure in Women With Gestational Diabetes Mellitus

**DOI:** 10.7759/cureus.35996

**Published:** 2023-03-10

**Authors:** Kok Wei Kan, Mohd-Alif Wan Mohd, Nik Lah Nik-Ahmad-Zuky, Ismail Shatriah

**Affiliations:** 1 Department of Ophthalmology and Visual Sciences, School of Medical Sciences, Universiti Sains Malaysia, Kubang Kerian, MYS; 2 Ophthalmology Clinic, Universiti Sains Malaysia (USM) Hospital, Universiti Sains Malaysia, Kubang Kerian, MYS; 3 Department of Obstetrics and Gynecology, School of Medical Sciences, Universiti Sains Malaysia, Kubang Kerian, MYS

**Keywords:** intraocular pressure, central corneal thickness, pregnant woman, diet control, gestational diabetes

## Abstract

Introduction: Pregnancy causes an increase in central corneal thickness (CCT) and a reduction in intraocular pressure (IOP), especially in the third trimester. However, there is very limited published data regarding CCT and IOP in gestational diabetes mellitus (GDM) on diet control. This study is aimed to compare the means of CCT and IOP between pregnant women with GDM on diet control, healthy pregnant women, and healthy non-pregnant women.

Methods: This is a comparative cross-sectional study. A total of 184 women were recruited and divided into the following three groups: 61 pregnant women with GDM on diet control, 63 healthy pregnant women, and 60 healthy non-pregnant women as control. All subjects have undergone ocular examination during their 36-40 weeks of gestation. CCT measurement was done using a specular microscope and IOP measurement using a non-contact tonometer. Data from the right eye were analyzed.

Results: The mean age was 32 (4.0) years in GDM on diet control, 29 (3.0) years in healthy pregnant women, and 27 (5.4) years in healthy non-pregnant women. The number of gravidas was 2.5 (0.8) in women with GDM on diet control and 2.3 (0.8) in healthy pregnant women. There was a significant difference (p<0.05) in the mean CCT in women with GDM on diet control compared to healthy pregnant and healthy non-pregnant women. The mean IOP is significantly lower in both pregnant women with GDM on diet control and healthy pregnant groups, compared to the healthy non-pregnant women group.

Conclusion: Women with GDM showed significantly thicker mean CCT than healthy pregnant and non-pregnant women. The mean IOP is significantly lower in both pregnant women with GDM on diet control and healthy pregnant groups, compared to the healthy non-pregnant women group.

## Introduction

Gestational diabetes mellitus (GDM) is defined as any degree of glucose intolerance with the onset or first recognition of pregnancy. The prevalence of GDM has been reported to vary substantially, ranging from 1% to 30% globally [1,2], 11.5% in Asia [3], and 10.07% in Eastern and Southeastern Asia [4]. A recent meta-analysis involving local data revealed that the prevalence of GDM in Malaysia was approximately 21.5% [5]. Pregnancy has been demonstrated to cause variable ocular tissue changes, including those relating to central corneal thickness (CCT) and intraocular pressure (IOP) [6-11].

Santiagu et al. reported a mean CCT in GDM during the third trimester and after postpartum [12]. Based on a PubMed search, we were unable to find any published articles that examined both CCT and IOP in pregnant women with GDM. This is important for the interpretation of increased IOP, especially in pregnant women with GDM, because the disease has a high prevalence rate in Malaysia. This study focuses on the comparison of mean CCT and IOP measurements in patients with GDM on diet control, healthy pregnant women, and healthy non-pregnant women.

## Materials and methods

This research concerns a cross-sectional study conducted in the Hospital Universiti Sains Malaysia from January 2015 to February 2017. Pregnant women with GDM on diet control, healthy pregnant women, and healthy non-pregnant women aged between 18 and 45 years were recruited. The study was conducted in accordance with the Declaration of Helsinki and approved by the Human Research Ethics Committee of the Universiti Sains Malaysia (USM/JEPeM/276.2.{2}). Written consent was obtained from the subjects.

Pregnant women who were at 36-40 weeks of gestation and attending routine antenatal checkups at our institution's Obstetrics and Gynecology Clinic were prospectively enrolled in the study. The inclusion criteria for pregnant women with GDM were women having an uncomplicated singleton pregnancy, a confirmed GDM diagnosis with 75 g oral glucose tolerance test, hemoglobin A1c (HbA1c) level of less than 6% at all times, aged between 18 and 45 years, and on diet control only. Healthy pregnant women were recruited from the same age group among women with no concurrent medical problems. Healthy non-pregnant women were recruited from among staff or volunteers. Women with known systemic disease or known ophthalmic disorder, contact lens wearers, women with a refractive error exceeding ± 3.0 diopters, and women who had undergone any type of previous ocular surgery were excluded from this study.

All subjects were screened for inclusion and exclusion criteria during an interview session. Written informed consent was obtained from all the participants. Demographic data collected included age, race, gravida, parity, duration of gestation, and family history of diabetes mellitus (DM). A complete ophthalmic examination was performed, including best-corrected visual acuity, refraction, anterior segment, IOP measurement, and fundus examination to look for signs of diabetic retinopathy.

Only data from the right eye were chosen and included in this study. The CCT was measured using the Topcon SP-2000P non-contact specular microscope (Tokyo, Japan: Topcon Inc.), which captures an image of the endothelium cells and measures cornea thickness; an average of three consecutive readings were used for data analysis.

The IOP was measured using the Reichert 7CR (Berwyn, PA: Ametek Inc.), which is a non-contact tonometer. It utilizes a patented bi-directional applanation process to characterize the biomechanical properties of the cornea and reduce their impact on the IOP measurement. The average of three consecutive readings with acceptable scores (less than 5) in auto mode was documented for data analysis. No topical agent was used prior to the IOP measurement. All measurements and examinations were performed by the principal investigator.

The demographic data, parity, clinical findings, CCT, and IOP were documented on a separate data collection sheet. The data were then analyzed using the SPSS version 22.0 (Armonk, NY: IBM Corp.). The means of the CCT and IOP among pregnant women with GDM on diet control, healthy pregnant women, and healthy non-pregnant women were compared using a one-way analysis of variance (ANOVA) test. A p-value less than 0.05 was considered statistically significant.

## Results

All 184 subjects in the three groups had the best corrected visual acuity of 6/6 (20/20). The anterior segment and fundus examinations indicated clear lens and normal funduscopic findings without diabetic retinopathy changes. Table 1 presents the demographic data for the three groups. The mean age for the group of women with GDM on diet control was 32 (4.0) years, 29 (3.0) years for the healthy pregnant women group, and 27 (5.4) years for the healthy non-pregnant women. Most of the subjects were of Malay ethnicity. The mean gravida was 2.5 (0.8) for women with GDM on diet control and 2.3 (0.8) for healthy pregnant women. A family history of DM was observed in 60.7% of women with GDM on diet control, 39.7% in healthy pregnant women, and 30% in healthy non-pregnant women.

**Table 1:** Demographic data of pregnant women with GDM, healthy pregnant women, and healthy non-pregnant women. SD: standard deviation; DM: diabetes mellitus; GDM: gestational diabetes mellitus; NA: not available

Variables		GDM on diet control (n=61)	Healthy pregnant women (n=63)	Healthy non-pregnant women (n=60)
Mean age (years) (SD)		32 (4.0)	29 (3.0)	27 (5.4)
Race (n, %)	Malay	60 (98.4)	61 (96.8)	57 (95.0)
	Chinese	1 (1.6)	2 (3.2)	3 (5.0)
Gravida (SD)		2.5 (0.8)	2.3 (0.8)	NA
Parity (SD)		1.3 (0.8)	1.1 (0.7)	0.5 (0.8)
Gestation (weeks) (SD)		37.9 (1.1)	37.6 (1.1)	NA
Family history of DM (n, %)	Yes	37 (60.7)	25 (39.7)	18 (30.0)
	No	24 (39.3)	38 (60.3)	42 (70.0)

Table 2 presents the comparison of the means of CCT and IOP for the studied groups. The mean CCT was 552.28 (22.59) μm in the group of women with GDM on diet control, 538.75 (22.92) μm in the healthy pregnant women, and 525.88 (19.31) μm in the healthy non-pregnant women. A statistically significant difference (p<0.05) was found in the mean CCT among the three groups. The mean IOP is significantly lower (p<0.05) in both pregnant women groups as compared to the healthy non-pregnant women group. The mean IOP was 12.92 (2.06) mmHg and 12.34 (2.58) mmHg for GDM on diet control and healthy pregnant women, respectively. Meanwhile, the mean IOP for healthy non-pregnant women was 14.20 (2.78) mmHg.

**Table 2:** Mean CCT and IOP in each group. One-way ANOVA (p<0.05 is significant). CCT: central corneal thickness; IOP: intraocular pressure; SD: standard deviation; GDM: gestational diabetes mellitus

Variables	GDM on diet control (n=61)	Healthy pregnant women (n=63)	Healthy non-pregnant women (n=60)	p-Value
Mean CCT (SD)	552.28 (22.59)	538.75 (22.92)	525.88 (19.31)	0.001
Mean IOP (SD)	12.92 (2.06)	12.34 (2.58)	14.20 (2.78)	<0.001

Table 3 presents a post hoc comparison of the means of CCT and IOP for the three groups. There was a statistically significant difference in the mean CCT among all three groups (p<0.05). Meanwhile, the comparison of the mean IOP indicates a significant difference between the women with GDM on diet control and healthy pregnant women against the non-pregnant group (p<0.05). However, there was no significant difference when comparing the parameter between women with GDM on diet control and healthy pregnant women (p>0.05).

**Table 3:** Post hoc comparison of mean CCT and IOP based on GDM on diet control. One-way ANOVA (p<0.05 is significant). CCT: central corneal thickness; IOP: intraocular pressure; SD: standard deviation; GDM: gestational diabetes mellitus

Comparison		n	Mean difference (95% CI)	F-statistics (df1, df2)	p-Value
Mean CCT	GDM-healthy pregnant	61	13.53 (3.91, 23.15)	22.398 (2, 181)	0.002
	GDM-healthy non-pregnant	63	26.40 (16.66, 36.13)		0.001
	Healthy pregnant-healthy non-pregnant	60	12.86 (3.20, 22.52)		0.003
Mean IOP	GDM-healthy pregnant	61	0.58 (-0.53, 1.68)	8.883 (2, 181)	0.401
	GDM-healthy non-pregnant	63	-1.28 (-2.40, -0.16)		0.015
	Healthy pregnant-healthy non-pregnant	60	-1.86 (-2.97, -0.75)		0.001

## Discussion

We present new data on the means of CCT and IOP in GDM women on diet control in a hospital-based study. Table 4 summarizes the published studies on GDM, healthy pregnant women, and non-pregnant women included in our study outcome. Our study describes both the means of CCT and IOP in GDM on diet control, healthy pregnant women, and non-pregnant women during the third trimester.

**Table 4:** Comparison of CCT and IOP during third trimester of healthy pregnant women and healthy women in previously published data. CCT: central corneal thickness; IOP: intraocular pressure; GDM: gestational diabetes mellitus; SD: standard deviation; USG: ultrasonography; NCT: non-contact tonometer; GAT: Goldman applanation tonometer

	Present study			Santiagu et al. [12]			Akar et al. [13]		Efe et al. [14]		Sundaram et al. [15]	
Country	Malaysia			Malaysia			Turkey		Turkey		India	
Year	2022			2017			2005		2012		2016	
Groups	GDM	Healthy pregnancy	Non-pregnant	GDM	Normal pregnancy	Pregestational diabetes	Pregnant	Non-pregnant	Pregnant	Non-pregnant	Pregnant	Non-pregnant
Number	61	63	60	71	70	51	88	94	25	NA	100	NA
Mean age (SD) in years	32 (4.0)	29 (3.0)	27 (5.4)	32.2	29.9	31.1	26.1 (2.2)	25.4 (2.3)	29.0 (3.0)	NA	25.0 (3.9)	NA
Mean CCT (SD)	552.2 (22.5)	538.7 (22.9)	525.8 (19.3)	540.0 (31.9)	540.7 (29.5)	543.2 (34.9)	NA	NA	573.7 (24.0)	NA	NA	NA
CCT method	Specular microscope			USG			NA		USG	NA	NA	NA
Mean IOP (SD)	12.9 (2.1)	12.3 (2.6)	14.2 (2.8)	NA	NA	NA	13.7 (2.2)	14.1 (2.1)	12.4 (2.1)	NA	11.1 (1.1)	NA
IOP method	NCT			NA			NCT		NCT	NA	GAT	NA

Our data indicate that the mean CCT is the thickest in women with GDM on diet control and thinnest in healthy non-pregnant women (p<0.05). Our results contradict the study by Santiagu et al., who reported no significant difference in the mean CCT among women with GDM, pre-gestational DM, and normal pregnancy [12]. They postulated that the phenomenon was due to strict control of diabetes during pregnancy [12]. Our patients documented HbA1c of less than 6% during the study recruitment, and we observed a different outcome.

Our findings suggest that pregnancy and transient hyperglycemia contribute to a thicker value of CCT during the third trimester in women with GDM on diet control. High estrogen levels during pregnancy, especially in the third trimester, cause stromal hydration and result in thicker CCT [10]. This is further evidenced by the presence of estrogen receptors in the nuclei of the stromal and endothelial cells of the cornea [16,17]. Another postulation of thicker CCT during pregnancy is due to the secondary effect of systemic water retention on the cornea by estrogen-induced upregulation of the renin-aldosterone system [17,18]. This was further demonstrated by the CCT returning to pre-pregnancy levels three months after delivery [7,8].

The thicker CCT value in pregnant women with GDM may also be due to excess glucose in the cornea, leading to the intracellular accumulation of sorbitol, acting as an osmotic agent and causing cornea cells to expand [19]. In addition, Del Buey et al. and Altay et al. have postulated that alterations in the corneal epithelium, stroma, and endothelium, in particular, accumulation of advanced end products from glycation and oxidation of proteins and lipids, result in an increase in CCT in diabetic patients [20,21]. Some authors have hypothesized that corneal endothelium dysfunction is also one of the factors causing increased corneal hydration, hence the thicker CCT [19,22,23].

Multiple published studies have reported that IOP is statistically lower in pregnant women compared to healthy non-pregnant women [24-26]. We noted similar findings in our study. Previous studies have postulated that IOP reduction can be due to various mechanisms. High progesterone levels during pregnancy can cause an increase in the fluid outflow from the eye and a reduction in the episcleral venous pressure resulting from decreased total systemic vascular resistance [16,18,24]. Wang et al. and Shrinkhal et al. have added that human chorionic gonadotrophin, nitric oxide, endothelin-I, eicosanoids, and relaxin also play a role in the reduction of IOP in pregnancy [9,24].

In this study, we highlighted no significant difference in the mean IOP when comparing the women with GDM on diet control with healthy pregnant women. Our finding is consistent with Kan et al., who reported no significant difference in IOP between GDM and healthy pregnant women [27]. We hypothesize that this may be due to early detection, strict control, and monitoring of GDM in our setting, which maintains the blood glucose levels close to that of healthy pregnant women. This is in contrast to a study by Pérez-Rico et al. that found IOP was significantly higher in diabetic patients compared to healthy subjects [28]. They attributed this to hyperglycemia-induced corneal thickening that may cause an IOP overestimation among diabetic patients.

We looked at CCT and IOP changes between 36 and 40 weeks of gestation since several studies have indicated that this is when the CCT and IOP change the most, compared to other periods of pregnancy [6,8,9]. Central corneal thickness has been known to influence IOP calculation using the Goldmann applanation tonometer (GAT); a thicker cornea will give a falsely higher value and vice versa. Variations in CCT would impact IOP measurements using GAT, as GAT was gauged for a CCT of 520 μm [29]. However, we found that the increased CCT in women with GDM on diet control had no impact on the IOP. We hypothesize that the newer generation of NCTs is better at eliminating corneal factors that influence IOP measurement. These changes may have an impact on the progression of a pre-existing ocular disease. More research is required to determine the variability of these measurements in patients with GDM throughout pregnancy.

## Conclusions

Both pregnancy and diabetes are associated with several ocular changes. Our study indicates that women with GDM have significantly thicker mean CCT compared to healthy pregnant and healthy non-pregnant women. The mean IOP is significantly lower in both pregnant women with GDM on diet control and healthy pregnant groups, compared to the healthy non-pregnant women group. The outcome of this study is expected to create awareness on ocular changes in women with GDM and facilitate early recognition and prompt treatment where necessary.
